# Weather effects on hand, foot, and mouth disease at individual level: a case-crossover study

**DOI:** 10.1186/s12879-019-4645-4

**Published:** 2019-12-03

**Authors:** Zhicheng Du, Shao Lin, Tia Marks, Wangjian Zhang, Te Deng, Shicheng Yu, Yuantao Hao

**Affiliations:** 1 Department of Medical Statistics and Epidemiology, School of Public Health, Sun Yat-sen University, Guangzhou, 510080 China; 2 Department of Environmental Health Sciences, School of Public Health, University at Albany, State University of New York, Rensselaer, New York, 12144 USA; 3 Healthcare Department, Nanshan Maternity & Child Healthcare Hospital of Shenzhen, Shenzhen, 518000 China; 4 Chinese Center for Disease Control and Prevention, Beijing, 102206 China

**Keywords:** Hand, foot, and mouth disease, Case-crossover, Weather effect

## Abstract

**Background:**

Hand, foot, and mouth disease (HFMD) raises an urgent public health issue in the Asia-Pacific region, especially in China. The associations between weather factors and HFMD have been widely studied but with inconsistent results. Moreover, previous studies utilizing ecological design could not rule out the bias of exposure misclassification and unobserved confounders.

**Methods:**

We used case-crossover analysis to assess the associations of weather factors on HFMD. Individual HFMD cases from 2009 to 2012 in Guangdong were collected and cases located within 10 km of the meteorological monitoring sites were included. Lag effects were examined through the previous 7 days. In addition, we explored the variability by changing the distance within 20 km and 30 km.

**Results:**

We observed associations between HFMD and weather factors, including temperature and relative humidity. An approximately U-shaped relationship was observed for the associations of temperature on HFMD across the same day and the previous 7 days, while an approximately exponential-shaped was seen for relative humidity. Statistically significant increases in rates of HFMD were associated with each 10-unit increases in temperature [Excess rate (ER): 7.7%; 95% Confidence Interval (CI): 3.9, 11.7%] and relative humidity (ER: 1.9%; 95% CI: 0.7, 3.0%) on lag days 0–6, when assessing within 10 km of the monitoring sites. Potential thresholds for temperature (30.0 °C) and relative humidity (70.3%) detected showed associations with HFMD. The associations remained robust for 20 km and 30 km.

**Conclusions:**

Our study found that temperature and relative humidity are significantly associated with the increased rates of HFMD. Thresholds and lag effects were observed between weather factors and HFMD. Our findings are useful for planning on targeted prevention and control of HFMD.

## Background

Hand, foot, and mouth disease (HFMD) is a pediatric infectious disease causing outbreaks worldwide [1]. At least in mainland China, HFMD was reported as the infectious disease with highest yearly incidence with a 9 year average (114.48 per 100,000) from 2004 to 2013 [2]. Although HFMD cases are often mild, severe and fatal cases among children are not rare [3]. HFMD has become a vital public health concern due to its threat to children and its huge burdens on society.

Weather factors (e.g., temperature and relative humidity) have been widely reported as having associations with HFMD in previous studies. Temperature was estimated to have a risk ratio of 1.30 (95% CI: 1.23, 1.37) at the 91st percentile compared to the 50th [4]. The commonly hot days were found to increase HFMD burdens with a relative risk peaking at around 6 days of lag [5]. One of our preceding studies also found that temperature (relative risk, RR: 1.039; 95% CI: 1.028, 1.050) and relative humidity (RR: 1.015; 95% CI: 1.010, 1.021) were statistically associated with HFMD incidence using a Bayesian spatiotemporal model [6].

Biases from exposure misclassification were major limitations in previous studies. For studies using a time-series design or other ecological designs, the mean value of weather factors from limited monitoring sites were usually assigned to all HFMD cases located throughout a large region no matter how far they lived from the monitor (e.g., a city or a province) [7–11]. Including cases farther away from monitoring sites and using the same values as those near the monitoring sites can result in an underestimation due to the bias of exposure misclassification or exposure error [12–15]. Moreover, these studies were analyzed at the population level (i.e., ecological design) [16], which usually assigns the mean value of weather factors to a group of subjects rather than a single subject. In addition, these studies might miss some important confounders at individual levels such as age, gender, and race/ethnicity.

To address these biases of exposure misclassification and unobserved confounders, we conducted this case-crossover study to examine the association between short-term (within 1 week) increases in weather factors and HFMD, weather factors’ lag effects (0–7 days) and potential thresholds on HFMD, and validate the exposure-health association by evaluating difference residential distances to monitor sites (10, 20, and 30 km).

## Methods

### Study design

To estimate the associations between HFMD and temperature and relative humidity, we used a case-crossover design and conditional logistic regression models [17]. The fixed 28-day-window case-crossover approach was used in this study and was recently used in previous studies of environmental health [18, 19]. Each HFMD case was defined as “case” in the matched case-control study. Each HFMD case in other weeks with the same calendar month and weekday when they did not get infection served as their own controls. Therefore, each HFMD case were compared to three or four controls within the same month. And the outcome in this study was a dichotomous variable with the “case” coded as “1” and the “control” coded as “0”. This design contrasts weather factors immediately before the HFMD cases onset to the weather factors of their own controls.

### Study population and HFMD cases data

Information on HFMD cases were obtained from the China Center for Disease Control and Prevention (China CDC), which we have used previously [3, 10]. HFMD has been included as a notifiable infectious disease since May 2008. Data on HFMD cases are collected in a legislatively mandated database, covering almost all cases in China [20]. HFMD data include patient information on the laboratory testing results and severity, as well as demographic characteristics including date of birth, gender, residential address, occupation, and onset information. Anyone who reports cases to the system must be strictly certified to ensure accuracy and completeness of data. We geocoded the residential address for all cases using the Geocoder service of Baidu Maps [21].

HFMD cases aged <18 years old with an onset date from January 1, 2009 to December 31, 2012 for residents of Guangdong were obtained. We then included all study subjects living within 10, 20, and 30 km (km) of any of the 36 weather monitoring sites in Guangdong (described below), leaving *N* = 100,669, 280,957, 490,497 available cases for analyses. Missing values in virus types and severity were recoded as “missing”. No missing data on other variables was observed for the HFMD cases data. This study was reviewed and approved by the Institutional Review Board at the School of Public Health, Sun Yat-Sen University.

### Weather data

**Fig. 1:**
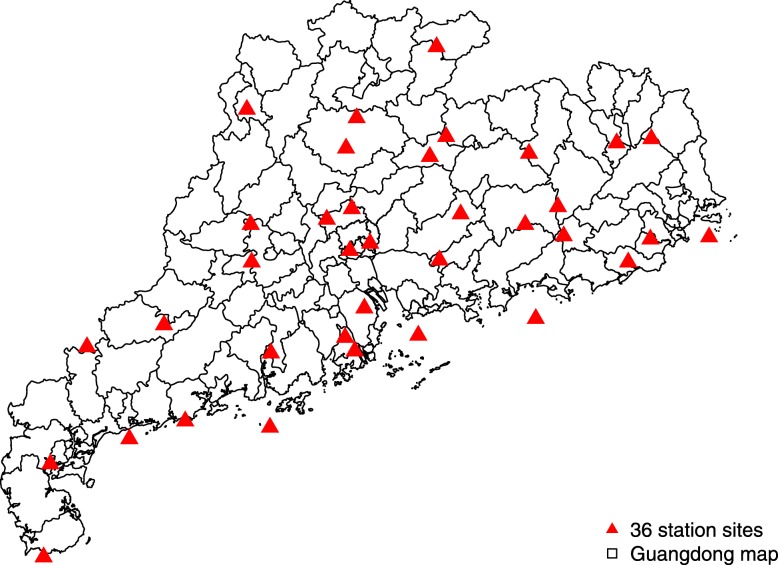
Locations of 36 national meteorological monitoring sites in Guangdong. This map was downloaded from OpenStreetMap (The cartography in the OpenStreetMap map tiles is licensed under CC BY-SA (www.openstreetmap.org/copyright, © OpenStreetMap contributor). The licence terms can be found on the following link: http://creativecommons.org/licenses/by-sa/2.0/) and processed by and R version 3.4.3 (R Core Team, Vienna, Austria, 2017, https://www.R-project.org)

Daily temperature and relative humidity data were retrieved from the National Meteorological Information Center (http://data.cma.cn/) for each of the 36 monitoring sites (Fig. 1) where measurements were collected (19 out of 21 cities in Guangdong): Shaoguan, Qingyuan, Heyuan, Meizhou, Zhaoqing, Guangzhou, Dongguan, Huizhou, Jieyang, Shantou, Maoming, Yunfu, Jiangmen, Zhongshan, Zhuhai, Shenzhen, Shanwei, Zhanjiang and Yangjiang. At each site, hourly temperature and relative humidity were measured using platinum resistance temperature detectors and capacitive thin-film polymer sensors, respectively. In our study, 24-h daily averages were used. We assigned the measurements of each monitoring site to the cases within 10, 20, and 30 km. Consistent with previous studies [5, 7, 18, 22], we calculated the mean of weather factors for the onset day (lag day 0), and previous 2 to 14 days (lag days 0–1 to 0–13) to estimate the cumulative lagged effects. Another reasoning for setting lag days is HFMD has an incubation period of 3–7 days [23]. No missing data was observed for the weather data.

### Statistical analyses

Using descriptive analyses, we assessed the distribution of case characteristics (sex, age, occupation, virus, and severity) by distance away from each meteorological monitoring site (within 10, 20, and 30 km).

Conditional logistic regression model was used to estimate the relative risk (RR) and 95% confidence interval (CI) of HFMD associated with each 10-unit increases in temperature and relative humidity during each lag period. The conditional logistic regression model can then be specified as below:

$$ \mathrm{logit}(p)=\beta X+{a}_{stratum(i)} $$

where, *p* is the probability of being a case. *X* is the weather factors (i.e., temperature or relative humidity). *β* is the regression coefficients, and they can be converted to RR by e^*β*^. *stratum (i)* indicates each case-control pair, and *α* is the stratum constant.

Moreover, we calculated the excess rate (ER) using (RR-1) × 100% to make it easier to interpret and compare. Piecewise linear regression [24] was used to identify the change points (thresholds at which the association between HFMD and weather factors change) in the association between HFMD and weather factors. We also conducted the subgroup analyses by sex, age, and occupation to estimate the ERs among different population groups on lag days 0–6 within 10 km. R version 3.5.1 (R Foundation for Statistical Computing, Vienna, Austria; https://www.r-project.org/) was used for all data managements and statistical analyses in this study.

## Results

Table 1 shows case characteristics, by distance away from monitoring sites. Of the 921,499 HFMD cases during the study period, 10.92% of cases were included within 10 km of the monitoring sites, 30.49% cases for 20 km, and 53.23% cases for 30 km. Across different distances, cases were primarily male (exceed 63%), 3 years old and younger (exceed 80%), home-care children (exceed 69%), and mild cases (exceed 99%). With the exception of the missing values, the viruses of Coxsackievirus A16 (CoxA16), Enterovirus 71 (EV71), and others showed similar values of proportions. However, the proportions of different characteristics showed statistical difference among different distances due to the large sample size.

**Table 1:** Cases characteristics and within different distance of the monitoring sites

	Total N (%)	10 km N (%)	20 km N (%)	30 km N (%)	*P* value*
Sample size	921,499 (100)	100,669 (10.92)	280,957 (30.49)	490,497 (53.23)	
Sex					0.016
female	327,858 (35.58)	36,770 (36.53)	101,648 (36.18)	176,843 (36.05)	
male	593,641 (64.42)	63,899 (63.47)	179,309 (63.82)	313,654 (63.95)	
Age					<0.001
(0, 1]	402,160 (43.64)	39,806 (39.54)	119,233 (42.44)	209,734 (42.76)	
(1, 3]	362,151 (39.30)	43,096 (42.81)	115,171 (40.99)	199,021 (40.58)	
(3, 5]	112,854 (12.25)	13,051 (12.96)	34,217 (12.18)	59,709 (12.17)	
(5, 18]	44,334 (4.81)	4716 (4.68)	12,336 (4.39)	22,033 (4.49)	
Occupation					<0.001
student	27,126 (2.94)	2844 (2.83)	7616 (2.71)	13,786 (2.81)	
preschool	182,051 (19.76)	27,330 (27.15)	64,607 (23.00)	111,652 (22.76)	
home-care	709,310 (76.97)	70,364 (69.90)	208,133 (74.08)	363,730 (74.16)	
others	3012 (0.33)	131 (0.13)	601 (0.21)	1329 (0.27)	
Virus					<0.001
CoxA16	6113 (0.66)	2345 (2.33)	4012 (1.43)	4806 (0.98)	
EV71	8207 (0.89)	1848 (1.84)	3747 (1.33)	5353 (1.09)	
others	7122 (0.77)	2843 (2.82)	4871 (1.73)	5840 (1.19)	
missing	900,057 (97.67)	93,633 (93.01)	268,327 (95.50)	474,498 (96.74)	
Severity					<0.001
mild	916,653 (99.47)	100,213 (99.55)	279,505 (99.48)	487,676 (99.42)	
severe	3737 (0.41)	364 (0.36)	1152 (0.41)	2244 (0.46)	
missing	1109 (0.12)	92 (0.09)	300 (0.11)	577 (0.12)	

*Chi-square test

Table 2 describes the weather factors using mean and median statistics. For weather factors without lags (lag day 0), temperature (mean of 10 km: 26.7 °C vs. 26.6 °C) of case periods were slightly higher than control periods, while the relative humidity (77% vs. 77.4%) was slightly lower during case periods than control periods. For weather factors with lag days 0–6, temperature had the same distribution as lag day 0, while the relative humidity was equal (77.4%) during both case and control periods. For weather factors with lag days 0–13, temperature had the same level (26.6 °C) between case periods and control periods, while the relative humidity (77.6% vs. 77.3%) was slightly higher during case periods than control periods.

**Table 2:** Distribution of weather factors for case periods and control periods

Lag	Weather factor	Distance	Case		Control	
			Mean ± SD*	Median (IQR*)	Mean ± SD	Median (IQR)
0	Temperature (°C)	10 km	26.7 ± 5.4	27.6 (23.8,30.4)	26.6 ± 5.5	27.5 (23.7,30.3)
		20 km	26.8 ± 5.6	27.7 (23.8,30.5)	26.7 ± 5.6	27.6 (23.7,30.4)
		30 km	26.9 ± 5.5	27.7 (23.9,30.5)	26.8 ± 5.6	27.7 (23.7,30.5)
	Relative humidity (%)	10 km	77 ± 11	78 (71,85)	77.4 ± 10.9	79 (71,85)
		20 km	76.7 ± 10.9	78 (70,84)	77.1 ± 10.8	78 (71,85)
		30 km	76.7 ± 10.9	78 (70,84)	77 ± 10.9	78 (71,85)
0–6	Temperature (°C)	10 km	26.7 ± 5	28 (23.9,29.9)	26.6 ± 5.1	28 (23.8,30)
		20 km	26.7 ± 5.1	28 (23.8,30.1)	26.7 ± 5.2	28 (23.8,30.1)
		30 km	26.8 ± 5.1	28.1 (23.9,30.1)	26.8 ± 5.1	28.1 (23.8,30.2)
	Relative humidity (%)	10 km	77.4 ± 8.2	78.6 (73.1,83.3)	77.4 ± 8.3	78.6 (72.9,83.3)
		20 km	77.2 ± 8.1	78.3 (72.7,83)	77 ± 8.3	78.1 (72.6,83)
		30 km	77.1 ± 8.2	78.3 (72.6,83)	77 ± 8.3	78.1 (72.4,83)
0–13	Temperature (°C)	10 km	26.6 ± 4.8	28 (23.9,30)	26.6 ± 4.9	28 (23.6,30)
		20 km	26.7 ± 4.9	28 (23.9,30.1)	26.7 ± 5	28 (23.6,30.2)
		30 km	26.8 ± 4.9	28.1 (23.9,30.2)	26.7 ± 5	28 (23.7,30.2)
	Relative humidity (%)	10 km	77.6 ± 7	78.9 (73.8,82.5)	77.3 ± 7.2	78.6 (73.3,82.4)
		20 km	77.3 ± 7	78.5 (73.2,82.3)	77 ± 7.2	78.1 (72.8,82.1)
		30 km	77.3 ± 7.1	78.4 (73.1,82.3)	76.9 ± 7.2	78 (72.6,82.1)

*SD Standard deviation, *IQR* Interquartile range

**Fig. 2:**
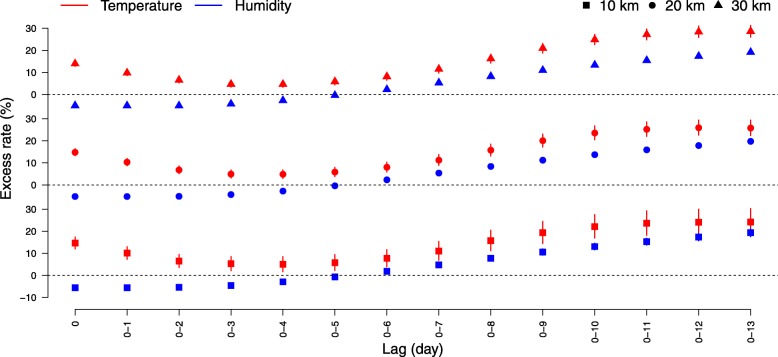
Excess rates of HFMD associated with each 10-unit increases in weather factors in Guangdong

Table 3 and Fig. 2 show the excess rates of HFMD associated with each 10-unit increases in weather factors by lag days. When assessing within 10 km of the monitoring sites, each 10-unit increases in temperature for all lag days were associated with 5.0–24.2% increased rates of HFMD. An approximately U-shaped relationship was observed for the relationship between HFMD and temperature. However, the excess rates of HFMD went up exponentially with each 10-unit increases in relative humidity across lag days, from − 5.6% (lag day 0) to 19.3% (lag days 0–13). Overall, statistically positive excess rates were found since lag days 0–6 for both weather factors. As seen in the sensitivity analyses, similar results were also observed for 20 km and 30 km. For the results of subgroup analyses by sex, age, and occupation (see Additional file 1: Table S1). We found that males, children younger than 1 year of age, and home-care children were more sensitive to the effect of temperature and relative humidity. In addition, an earlier effect was observed among the serotype-specific analyses with the limited number of cases (see Additional file 1: Table S2).

**Table 3:** Excess rates of HFMD associated with each 10-unit increases in weather factors

	10 km		20 km		30 km	
Lag day	Temperature (°C)	Relative humidity (%)	Temperature (°C)	Relative humidity (%)	Temperature (°C)	Relative humidity (%)
	ER (95% CI)*	ER (95% CI)	ER (95% CI)	ER (95% CI)	ER (95% CI)	ER (95% CI)
0	14.6 (11.9,17.4)	−5.6 (−6.3,-4.9)	14.8 (13.2,16.4)	−5.2 (−5.7,-4.8)	13.9 (12.8,15.2)	−5.0 (−5.3,-4.7)
0–1	10.1 (7.2,13.0)	−5.6 (−6.3,-4.8)	10.3 (8.6,12.1)	− 5.2 (− 5.7,-4.8)	9.8 (8.5,11.1)	− 5.1 (− 5.4,-4.7)
0–2	6.5 (3.5,9.6)	− 5.4 (− 6.2,-4.6)	6.8 (5.0,8.6)	− 5.2 (− 5.6,-4.7)	6.5 (5.2,7.9)	−5.1 (− 5.5,-4.7)
0–3	5.3 (2.1,8.6)	−4.6 (− 5.5,-3.7)	4.9 (3.1,6.8)	−4.3 (−4.9,-3.8)	4.7 (3.3,6.1)	−4.3 (−4.7,-3.9)
0–4	5.0 (1.6,8.5)	−2.9 (−3.9,-2.0)	4.9 (2.9,6.9)	−2.8 (−3.3,-2.2)	4.7 (3.2,6.2)	−2.7 (−3.2,-2.3)
0–5	5.8 (2.2,9.5)	−0.7 (−1.7,0.4)	5.9 (3.7,8.0)	−0.4 (−1.0,0.3)	5.8 (4.3,7.4)	−0.4 (−0.9,0.1)
0–6	7.7 (3.9,11.7)	1.9 (0.7,3.0)	8.0 (5.8,10.4)	2.3 (1.7,3.0)	8.1 (6.4,9.9)	2.3 (1.8,2.8)
0–7	11.0 (6.8,15.4)	4.8 (3.6,6.0)	11.3 (8.8,13.8)	5.4 (4.7,6.2)	11.5 (9.6,13.4)	5.3 (4.7,5.9)
0–8	15.7 (11.1,20.5)	7.7 (6.4,9.1)	15.8 (13.0,18.5)	8.4 (7.6,9.2)	16.2 (14.2,18.3)	8.2 (7.6,8.8)
0–9	19.3 (14.4,24.5)	10.6 (9.1,12.0)	20.1 (17.1,23.1)	11.2 (10.4,12.1)	21.0 (18.8,23.2)	11.0 (10.3,11.6)
0–10	22.1 (16.9,27.6)	13.1 (11.5,14.6)	23.6 (20.4,26.8)	13.7 (12.8,14.7)	24.9 (22.5,27.3)	13.4 (12.7,14.1)
0–11	23.6 (18.1,29.3)	15.3 (13.6,16.9)	25.2 (21.9,28.6)	15.9 (14.9,16.9)	27.2 (24.7,29.8)	15.4 (14.7,16.2)
0–12	24.1 (18.5,30.1)	17.3 (15.6,19.1)	25.9 (22.5,29.4)	17.9 (16.8,18.9)	28.4 (25.8,31.1)	17.3 (16.5,18.1)
0–13	24.2 (18.4,30.3)	19.3 (17.5,21.2)	25.8 (22.3,29.4)	19.8 (18.7,20.9)	28.6 (25.9,31.4)	19.1 (18.3,20.0)

*ER Excess rates, *CI* Confidence interval

**Fig. 3:**
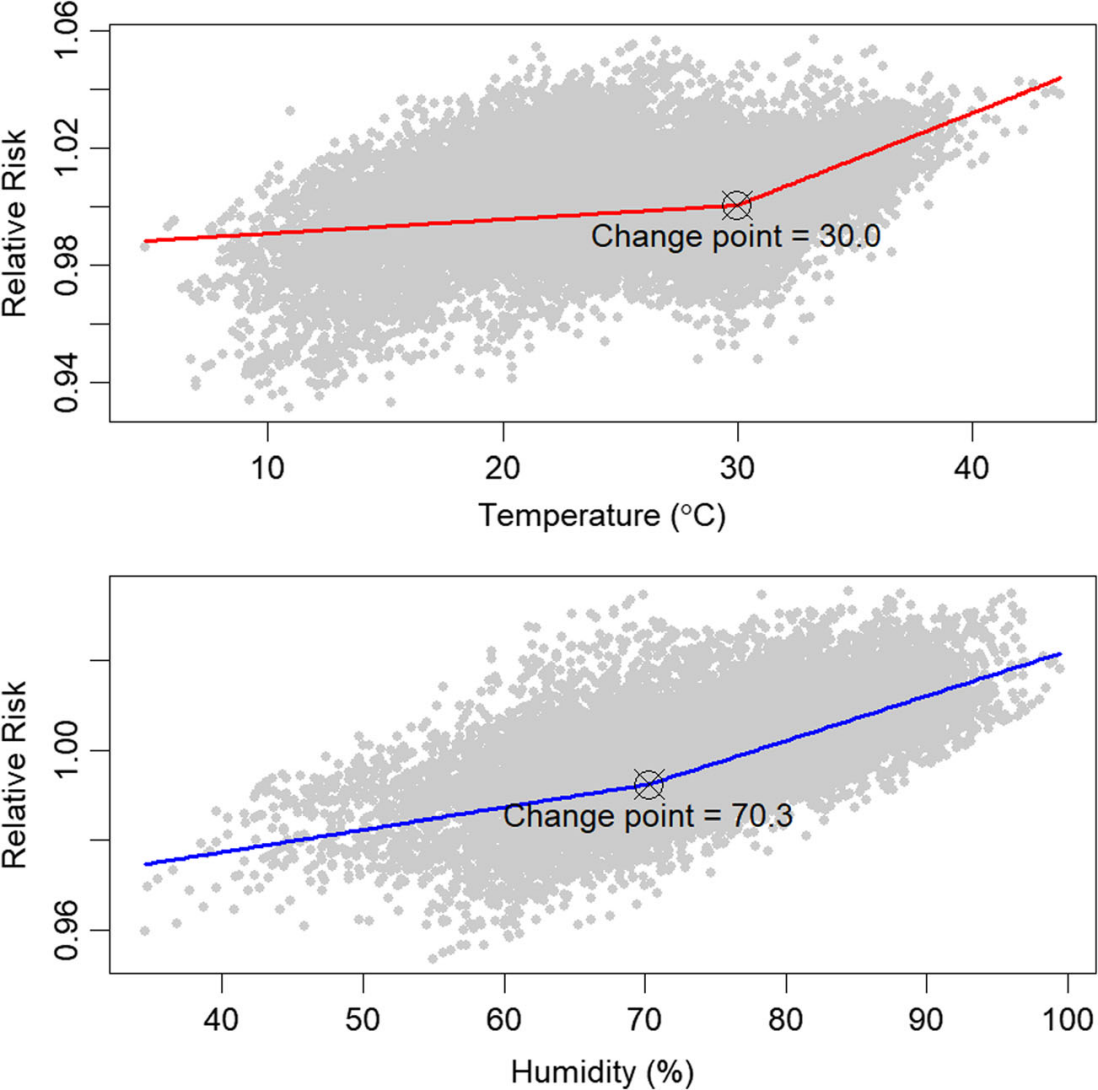
Change point detection in association between HFMD and weather factors in Guangdong

Figure 3 shows the change point of association between HFMD and weather factors on lag days 0–6 within 10 km of the monitoring sites. The RR of HFMD increased with the range of temperature and relative humidity. The change points for temperature and relative humidity were 30.0 °C and 70.3%, respectively. Different slopes were found before and after the change points, and the later slopes were more precipitous.

## Discussion

Using a case-crossover design, the present study included 0.2–0.7 million HFMD cases determined by distances away from monitoring sites through January 2009 to December 2012, accounting for 11–53% of reported cases in Guangdong. Similar demographic characteristics of cases across different distances away from monitoring sites indicated a random spatial distribution of HFMD cases, which showed the representativeness of the study population. While relative humidity was higher during control periods across different distances away from monitoring sites, temperature was higher during case periods. Interquartile range increases in temperature and relative humidity imposed statistically significant excess rates on HFMD. Increased rates of 4.7 (95%CI: 2.4, 7.1) for temperature and 1.9 (95% CI: 0.8, 3.1) for relative humidity were associated with HFMD on lag days 0–6 within 10 km of the monitoring sites.

For the study population, the case characteristics were consistent with previous studies [3, 25]. Male children aged ≤3 years old, and cared for at home tend to have higher infection rates. For the distribution of weather factors, our study differed from the previous studies by not reporting the weather by months, years, or even the whole study period [7, 26]. Our study was the first to describe the weather factors by case periods and control periods using a case-crossover design.

For the effects of temperature, our study validated the results of previous studies. We found all excess rates of temperature across lag days were positive (3.0–9.6%). One of our prior studies using Bayesian spatiotemporal model also reported that temperature (RR: 1.039; 95% CI: 1.028, 1.050) was statistically associated with HFMD [6]. The study by Onozuka et al. revealed that every 1 °C increase in average temperature increased by 11.2% for the weekly number of HFMD cases [27]. Higher temperature potentially enhances virus activity of HFMD and increases outdoor activities of children [9, 28].

For the effects of relative humidity, we found excess rates of relative humidity across lag days increased from a negative association (− 7.7%) to a positive association (1.9%). Similar trends were also observed in a time-series study using negative binomial regression [27] and another study which used distributed lag non-linear model [29]. Another study by Wu et al. found a negative effect of relative humidity at lag 1 day and positive effects on lag 5–7 days [30]. Besides the evidence of 3–7 days of incubation period for HFMD, it may take time for an environment with higher relative humidity to culture enteroviruses and children’s outdoor activities have the potential to be minimized during days with higher rainfall. Therefore, the positive effect on lag days 0–6 was more biologically plausible for both temperature and relative humidity.

For the subgroup analyses, we found males, children younger than 1 year of age, and home-care children were more sensitive to the effect of temperature and relative humidity. Consistent findings were also reported by one previous study [30]. More environment exposure and lower level of immunity for this susceptible group might be the potential reasons to cause a disease.

For the threshold effects, we detected change points for temperature (30.0 °C) and relative humidity (70.3%) on HFMD. Which means more attention should be paid to temperature and relative humidity change points and strengthened early warning are an urgent need for susceptible population. A study by Hii et al. found higher risk of HFMD in maximum temperature above 32 °C and in rainfall above 75 mm [9]. One of the previous studies also reported the threshold effects of weather factors on HFMD in China, with the temperature greater than 24.85 °C and the relative humidity between 80.59 and 82.55% [31]. Although different values were observed, both studies suggested higher temperature and higher relative humidity impose higher risk on HFMD.

For different distances of monitoring sites, we found a slightly increased rate of HFMD with both temperature and relative humidity from 10 to 30 km. The further one was supposed to have more bias from exposure misclassification. So, our study showed that the associations would be overestimated if the weather factors were assigned without considering the distances of cases from the monitoring sites.

The present study stood out from prior studies by its strengths. First, this is the first study to estimate the weather effects on HFMD at the individual level using a case-crossover design, which can inherently control for demographic characteristics. Second, we limited the cases within a certain distance of monitoring sites to reduce bias from exposure misclassification.

However, several limitations of this study should also be acknowledged. First, a limited number (i.e., thirty-six) of meteorological monitoring sites were available, resulting in an undercounting of the cases in our study due to further distance than 30 km. However, the distance of 30 km had included a large sample with 53.23% cases and the consistent findings from different distances (i.e., the statistically significant ERs were observed for both temperature and relative humidity since lag days 0–6) suggested that the effects of weather factors on HFMD will hardly change with the distance covering more cases. Second, the validity of HFMD reporting is usually a major concern in most vector-borne disease research. However, as HFMD data is a legislatively mandated database and all reporting persons have special certification, the likelihoods of inaccuracy and under-reporting are relatively small. Third, we didn’t include tropical cyclones and air pollution due to the availability of the data. Further studies incorporating the multiple factors are needed. However, previous studies have suggested that the effects of air pollution on health are much lower than the effects of weather factors [32, 33]. And the relationship we observed between HFMD and the weather factors might be not substantially confounded by the air pollution. Finally, although we observed a significant association between HFMD and weather factors, the pathogenic mechanism still cannot be well understood.

## Conclusions

Our study contributes to the limited knowledge of quantifying weather effects on HFMD at the individual level using a case-crossover design. Increased rates of HFMD were associated with increases in weather factors including temperature and relative humidity. Change points and lag effect were observed between weather factors and HFMD. Our study can serve as a reference for studying associations between diseases and environmental factors, and our findings are useful for targeted prevention and control of HFMD.

## Supplementary information

**Additional file 1: Table S1.** Excess rates of HFMD associated with each 10-unit increases in weather factors by demographics on lag days 0–6 within 10 km. **Table S2.** Excess rates of HFMD associated with each 10-unit increases in weather factors by serotypes and lag days within 10 km.

## Data Availability

All weather data we used are available as an open source and can be acquired according to the provenance listed in the “Methods” section (http://data.cma.cn/). The cases data are available from China CDC (http://www.phsciencedata.cn/Share/en/index.jsp), which were used under license and not publicly available.

## Abbreviations

CI: Confidence interval
ER: Excess rates
HFMD: Hand, foot, and mouth disease
IQR: Interquartile range
SD: Standard deviation
