# Supplementary material for: Weather effects on hand, foot, and mouth disease at individual level: a case-crossover study
Source: BMC Infect Dis. 2019 Dec 3;19:1029. doi: 10.1186/s12879-019-4645-4 (PMC6891988; doi:10.1186/s12879-019-4645-4)
Supplement: Supplementary file 1 — Additional file 1: Table S1. Excess rates of HFMD associated with each 10-unit increases in weather factors by demographics on lag days 0–6 within 10 km. Table S2. Excess rates of HFMD associated with each 10-unit increases in weather factors by serotypes and lag days within 10 km. [file 12879_2019_4645_MOESM1_ESM.docx]

**Table S1. Excess rates of HFMD associated with each 10-unit increases in weather factors by demographics on lag days 0-6 within 10km**

|  | Temperature (°C) | Relative humidity (%) |
| --- | --- | --- |
|  | ER (95%CI) | ER (95%CI) |
| Sex |  |  |
| male | 9.4 (4.5,14.6) | 2.6 (1.2,4.1) |
| female | 4.8 (-1.3,11.4) | 0.5 (-1.3,2.4) |
| Age |  |  |
| (0, 1] | 10.6 (4.3,17.2) | 2.9 (1.1,4.8) |
| (1, 3] | 4.1 (-1.6,10.0) | 3.8 (2.0,5.5) |
| (3, 5] | 6.1 (-4.3,17.7) | -2.9 (-5.9,0.1) |
| (5, 18] | 24.8 (4.9,48.4) | -10.6 (-15.1,-5.8) |
| Occupation |  |  |
| student | 14.0 (-8.3,41.8) | -14.1 (-19.6,-8.2) |
| preschool | 1.2 (-5.7,8.7) | 2.1 (-0.0,4.2) |
| home-care | 10.0 (5.3,14.9) | 2.4 (1.1,3.8) |
| others | 9.1 (-57.8,181.9) | 25.5 (-10.4,76.0) |

*ER: excess rates; CI: confidence interval

**Table S2. Excess rates of HFMD associated with each 10-unit increases in weather factors by** **serotypes and lag days within 10km**

|  | Temperature (°C), ER(95%CI) | | | Relative humidity (%), ER(95%CI) | | |
| --- | --- | --- | --- | --- | --- | --- |
| lag days | CoxA16 | EV71 | others | CoxA16 | EV71 | others |
| 0 | 17.9(0.1,38.8) | 24.2(4,48.2) | 28.2(10.1,49.3) | -10.8(-15,-6.5) | -8.9(-13.7,-3.8) | -6.9(-10.9,-2.6) |
| 0_1 | 19(-0.8,42.7) | 22.4(0.6,49.1) | 23.1(4,45.6) | -12.5(-16.9,-7.9) | -11.6(-16.6,-6.2) | -6.8(-11.2,-2.1) |
| 0_2 | 21.3(-0.4,47.8) | 22.2(-1.4,51.4) | 16.4(-3,39.7) | -13.5(-18.1,-8.6) | -14(-19.3,-8.4) | -6.6(-11.4,-1.6) |
| 0_3 | 17.2(-5,44.6) | 23.4(-1.8,55.2) | 16.6(-4.1,41.8) | -13.2(-18.2,-7.9) | -14.7(-20.2,-8.8) | -6(-11.1,-0.6) |
| 0_4 | 12.7(-9.6,40.4) | 19.7(-6.1,52.6) | 18.2(-4,45.4) | -13.5(-18.8,-7.9) | -14(-20,-7.7) | -4.2(-9.8,1.7) |
| 0_5 | 7.8(-14.3,35.7) | 17.3(-9.2,51.5) | 17.3(-5.7,46) | -13.8(-19.5,-7.8) | -12.6(-19,-5.7) | -2.6(-8.7,3.8) |
| 0_6 | 3.1(-18.8,30.9) | 18(-9.9,54.5) | 12.4(-10.7,41.4) | -13.7(-19.6,-7.3) | -10.3(-17.3,-2.7) | -1.3(-7.8,5.7) |
| 0_7 | 3.5(-19.3,32.7) | 21.4(-8.7,61.5) | 12.6(-11.6,43.4) | -12.4(-18.7,-5.6) | -6.7(-14.4,1.7) | 0(-7,7.5) |
| 0_8 | 3.6(-20,34.2) | 25.3(-7.1,69) | 16.6(-9.4,50.1) | -10.2(-17,-2.8) | -3.1(-11.5,6.1) | 0.4(-6.9,8.4) |
| 0_9 | 3.8(-20.6,35.5) | 33(-2.6,81.5) | 16.6(-10.2,51.5) | -8.1(-15.3,-0.2) | -0.2(-9.3,9.7) | 1.8(-6,10.3) |
| 0_10 | 3.2(-21.7,35.9) | 38.6(0.4,91.2) | 13.8(-13.2,49.1) | -6.1(-13.8,2.3) | 2.3(-7.4,13.1) | 3.2(-5.1,12.2) |
| 0_11 | 2.4(-22.9,36.1) | 42.9(2.4,99.3) | 10.8(-16.2,46.6) | -3.6(-11.9,5.4) | 5.2(-5.2,16.8) | 4.6(-4.2,14.2) |
| 0_12 | -0.7(-25.9,33.1) | 42.4(1.1,100.5) | 5.9(-20.6,41.3) | -0.5(-9.4,9.2) | 8.4(-2.8,20.8) | 6.1(-3.2,16.4) |
| 0_13 | -3.3(-28.4,30.5) | 42.3(0.1,102.3) | 2.2(-24,37.4) | 2.8(-6.8,13.4) | 11.2(-0.7,24.6) | 7.6(-2.3,18.5) |

*ER: excess rates; CI: confidence interval; CoxA16: Coxsackievirus A16; EV71: Enterovirus 71
